# Aerodynamic Performance of an Adaptive GFRP Wind Barrier Structure for Railway Bridges

**DOI:** 10.3390/ma13184214

**Published:** 2020-09-22

**Authors:** Yiqing Dai, Xuewei Dai, Yu Bai, Xuhui He

**Affiliations:** 1 Department of Civil Engineering, Monash University, Clayton, VIC 3800, Australia; Yu.Bai@monash.edu; 2 School of Civil Engineering, Central South University, Changsha 410075, China; 154811068@csu.edu.cn (X.D.); xuhuihe@csu.edu.cn (X.H.)

**Keywords:** fibre-reinforced polymer, wind barrier, railway bridge, aerodynamic coefficient, wind tunnel experiment, aerodynamics

## Abstract

Wind barrier structures on railway bridges are installed to mitigate the wind effects on travelling trains; however, they cause additional wind loads and associated aerodynamic effects on the bridge. An innovative concept was developed for a wind barrier structure in this study that used a glass–fibre–reinforced polymer (GFRP) that may deform properly when subjected to a crosswind. Such deformation then allows for wind to pass, therefore reducing the wind loads transferred to the bridge. Wind tunnel experiments were conducted on a 1/40-scale train and bridge models with the proposed GFRP barrier subjected to airflow at different speeds up to 20 m/s. The side-force and overturning-moment coefficients of both the train and the bridge were evaluated to characterise the aerodynamic effects. The results show that favourable side-force and overturning-moment coefficients of the train were provided by wind barriers taller than 10 cm. The aerodynamic coefficients of the train were not significantly affected by the airflow speeds; meanwhile, the overturning-moment coefficient of the bridge decreased with the increase in airflow speed due to smaller wind resistance of the barrier after deformation. A numerical analysis was conducted on both the reduced- and full-scale models of the train–barrier–bridge system and the results supported the findings obtained from the wind tunnel experiments.

## 1. Introduction

Strong crosswinds may cause safety problems, such as shaking, derailment and overturning of travelling trains [1,2,3]. Apart from the limitation on the operating speed and the cancellation of train trips in strong-wind scenarios, wind barriers have also been used to mitigate the negative effects of crosswind on trains [3,4], and are considered an effective and economical way [5,6] to improve the safety of travelling trains. However, for the bridges, this approach may in return cause additional wind loads associated with the aerodynamic effects acting on the wind barriers, which are transferred to the bridge. In order to ensure the safety of both travelling trains and the bridge, it may be required for the wind barrier to allow more or less wind to pass depending on the wind pressure level such that the wind load transferred to the structure can be reduced.

Wind tunnel tests [6,7,8,9] and numerical modelling [10,11,12,13] have been conducted to investigate the associated parameters to understand the aerodynamic performance of wind barriers and trains. For example, the effects of airflow speed, train speed and wind directions on the aerodynamic performance of a train with a rigid wind barrier were investigated through wind tunnel tests. It was concluded that a scenario with a static train is more critical than those with moving trains in terms of the mechanical loads on the train caused by the crosswind; meanwhile, the effect of airflow speeds on the aerodynamic coefficients of the train is insignificant [6]. In order to investigate the effect of the relative angle between the crosswind and the train (i.e., the yaw angle) on the mechanical responses of the train–barrier–bridge system, a numerical model of a railway system supported by a steel truss bridge was established using a computational fluid dynamics (CFD) method, where the simulation results were compared with those from wind tunnel experiments. It was suggested that airflow with a yaw angle of 90° (i.e., perpendicular to the travelling direction of the train) would lead to the maximum side force and overturning moment on the train [13]. 

According to the results from wind tunnel experiments on a train–barrier–bridge system [14,15], a taller wind barrier with a lower porosity may provide a greater reduction in the mechanical load on the train caused by the crosswind, and therefore provide better shielding effects; however, the side force and overturning moment transferred to the bridge from the wind barrier are increased. Therefore, the design of wind barriers (e.g., porosity and height) can introduce different effects on the aerodynamic performance of trains and bridges. In strong wind conditions, where train trips may be cancelled, or even during the time there are no trains travelling on the bridge, such shielding effects become unfavourable since adverse wind loads and the associated aerodynamic effects would be acting on the bridge. Several railways are supported on bridges [8]; therefore, the safety consideration for both travelling trains and supporting bridges with wind barriers needs to be addressed. Recently, louver-type wind barriers with adjustable blades were introduced [10,16], where the porosity of the wind barriers can be adjusted by changing the incline angle of the blades. For example, during regular conditions (i.e., without a strong crosswind), the wind barriers can be given a low porosity (achieved by putting the blades in closed mode) and better shielding effects can be provided to mitigate the wind effects on the train; meanwhile, during the presence of a strong crosswind, where the train trips are cancelled or during their intermission, the wind barriers can be adjusted to have high porosity (i.e., by opening the blades) and therefore cause a lower load to be transferred to the bridge. Obviously, such louver-type wind barriers require adjustment of the blades, where this would become time-consuming and hard to operate if completed manually, or would have a high cost and may not be reliable in harsh weather conditions (such as a strong wind) if completed automatically.

In this study, an innovative concept of a wind barrier structure to achieve such an adjustment that allows wind to pass was proposed using an adaptive method. This was done by using a wind barrier with an appropriate bending stiffness. When subjected to a strong crosswind, the wind barrier may deform as bending due to the transverse wind load, where the deformed wind barrier will naturally let wind pass through the deformed shape. Traditional wind barriers made from steel and concrete are associated with a high bending stiffness and the deformation when subjected to transverse wind is minor. In this study, glass-fibre-reinforced polymer (GFRP) composites were proposed for such wind barriers, where their elastic moduli ranged from 5 to 35 GPa [17,18], resulting in much lower structural stiffness than steel and reinforced concrete. Such GFRP composites have been introduced for the construction of railway noise barriers [19] and bridge decks [20]. However, the concept of a wind barrier that can allow wind to pass in an adaptive manner using deformation has not been examined yet, especially because such deformation caused by a wind load is associated with complex aerodynamic behaviour under different wind conditions. 

Experimental and numerical studies were conducted on the aerodynamic responses of a train–barrier–bridge system under a crosswind, where GFRP composites were used as the material for the wind barrier. In the wind tunnel experiments, a scale ratio of 1:40 was used for the models of the bridge and the train, as was used in [16] for a better comparison between the results of the proposed wind barrier and existing barrier types. A lateral crosswind at different speed levels was applied perpendicularly to the train–barrier–bridge system. The barrier heights ranged from 0 cm (no barrier) to 13.5 cm. The influences of the barrier height, airflow speed and location of the train on the aerodynamic responses of the system were studied. Based on the parameters of the airflow in the wind tunnel and the dimensions of the experimental models and the wind tunnel, both the reduced- and full-scale finite element (FE) models of the train–barrier–bridge system were established using finite element approaches, and the results were compared with the experimental results. The variations in the results from reduced- and full-scale models were also evaluated to validate the effectiveness of the reduced-scale experiments for predicting the aerodynamic behaviours of full-scale applications.

## 2. Material Properties of GFRP

GFRP materials have been used in bridge superstructures [21] and building structures [22,23,24] for their superior resistance to corrosion, high strength-to-weight ratio and competitive cost [17]. In this study, the wind barriers were made of GFRP plates with a thickness of 0.6 mm, which was chosen by considering the reduced scale and the bending stiffness requirements. The aerodynamic performance of the adaptive wind barrier is closely related to the elastic modulus of the plates, thus tensile tests on the GFRP plates were conducted.

**Figure 1:**
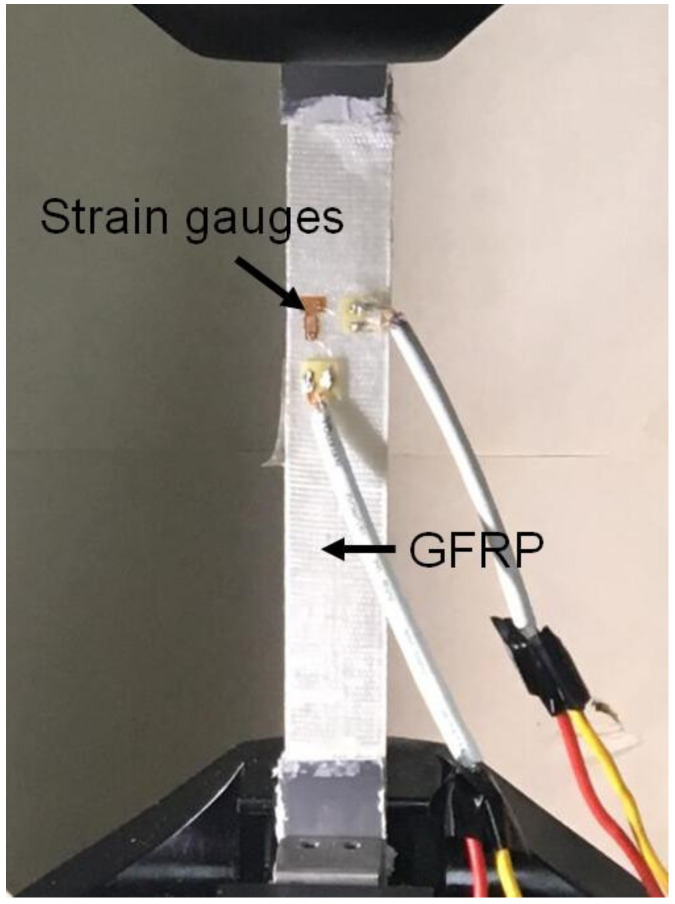
Tensile tests for the glass-fibre-reinforced polymer (GFRP) composite materials.

The GFRP plate was prepared through a wet layup approach using epoxy resin and two layers of woven GFRP fabrics, resulting in a total thickness of 0.6 mm. Five sample specimens with dimensions of 250 × 25 mm^2^ were cut from the same GFRP plate that was used for the wind barrier in the wind tunnel tests. Aluminium alloy plates with a length of 50 mm each were adhesively bonded to both ends of each GFRP specimen to protect the clamping area at both ends. As shown in Figure 1, two perpendicular strain gauges were installed at the centre of each GFRP specimen to obtain the strains in the longitudinal and transverse directions under loading. Tensile tests were conducted according to ASTM D3039 [25] for the five specimens. The load was applied using an MTS machine (MTS, Eden Prairie, MN, USA) at a loading rate of 2 mm/min until the failure of the specimens. The tensile loads and strain results were continuously recorded at 2 Hz during the loading process. 

The ultimate stress of the GFRP specimens was obtained from their ultimate loads and nominal cross-sectional areas (i.e., 25 × 0.6 mm^2^). The Poisson’s ratio was obtained based on the strain gauge readout in the longitudinal and transverse directions, which was determined to be 0.183 on average. The ultimate stress was determined to be 216.6 MPa on average and the average elastic modulus was obtained as 8.6 GPa based on the linear elastic responses.

## 3. Wind Tunnel Experiments

### 3.1. Experimental Setup and Procedure 

**Figure 2:**
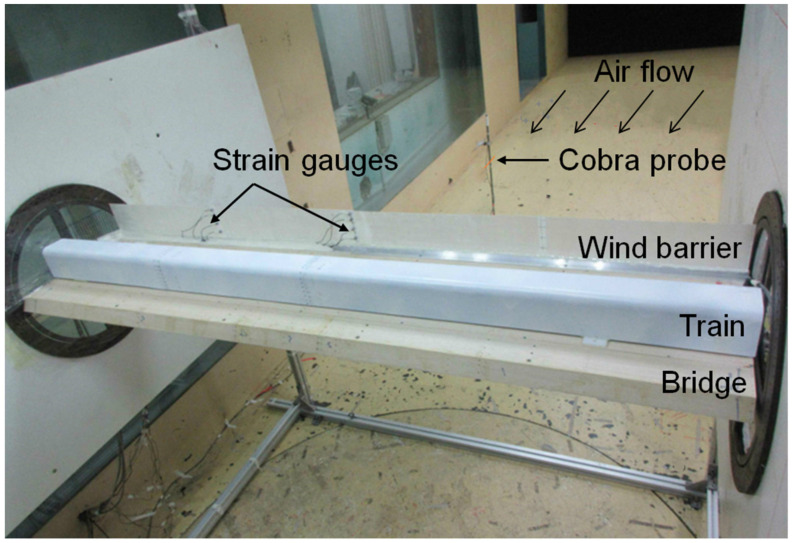
Wind tunnel experiments for the proposed barrier–train–bridge system.

The experiments were conducted in the wind tunnel of the National Engineering Laboratory for High-Speed Railway Construction at Central South University. The dimensions of the tunnel were 3 × 3 × 15 m (width × height × length). As shown in Figure 2, the train model made from polyvinyl chloride (PVC) was supported on a bridge model made from wood, while the GFRP barrier was installed on the bridge. The scale ratio was 1:40 for all the experimental models of the bridge, train and wind barriers, leading to a length of 2 m for both the train model and bridge section used in the tests. Wind barriers with five different heights (i.e., 0, 8, 10, 12 and 13.5 cm) were tested individually. The bottom end of the barrier was fixed on the bridge and the top was a free end, as shown in Figure 2. In the wind tunnel tests, three different scenarios of the trains were considered, including the absence of the train, a train on the windward side and a train on the leeward side. For each barrier height and train location, seven airflow speed levels were applied, ranging from 5 to 20 m/s; therefore, a total of 105 scenarios were examined. In each scenario, aerodynamic coefficients, including the side-force and overturning-moment coefficients of the train and bridge were obtained and used to evaluate the shielding effects of the wind barriers.

**Figure 3:**
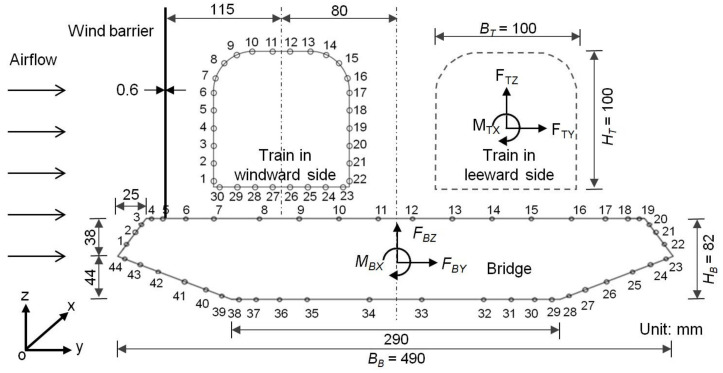
Numbering of the pressure measurement positions in the cross-sections of the bridge and the train and their geometries in the wind tunnel experiments (units in mm).

In the wind tunnel tests, the incoming airflow speed (*U*) was measured using a cobra probe (TFI Series 100, Turbulent Flow Instrumentation Pty, Tallangatta, VIC, Australia), as shown in Figure 2, at a frequency of 2000 Hz, where the measurement in each experimental scenario lasted for 20 s. The strains of the barriers were monitored using the strain gauges at key locations, as shown in Figure 2, and the deformation at the free end was also recorded. The wind pressures on the exterior surfaces of the train and bridge were measured using an electronic pressure scanner system (DSM 3400, Scanivalve, Liberty Lake, WA, USA). As shown in Figure 3, for each cross-section of the bridge model, 44 positions were subjected to a pressure measurement; meanwhile, 30 positions were monitored in each cross-section of the train model. A total of three cross-sections in the bridge model and two cross-sections in the train model were measured, leading to a total of 192 wind pressure measuring points. The total side force (*F_BY_*), lift force (*F_BZ_*) and overturning moment (*M_BX_*) on the bridge, as shown in Figure 3, were therefore calculated based on the pressure and the corresponding surface area of the bridge, as well as the actions transferred from the wind barriers (as derived from its deformation). The coordinate system is also shown in Figure 3, where the subscript “*B*” denotes the bridge. The total side force (*F_TY_*), lift force (*F_TZ_*) and overturning moment (*M_TX_*) on the train were also defined and calculated accordingly, where the subscript “*T*” denotes the train. 

### 3.2. Aerodynamic Coefficients

According to existing standards and References [4,5], several dimensionless aerodynamic coefficients of the train that were based on its side force (*F_TY_*), lift force (*F_TZ_*) and overturning moment (*M_TX_*) were used to evaluate the shielding effect of the wind barriers. These aerodynamic coefficients included the side-force coefficient (*C_TY_*), lift coefficient (*C_TZ_*) and overturning-moment coefficient (*C_TM_*), which were calculated for the train as follows:

(1) $$C_{TY}=\frac{F_{TY}}{0.5\rho U^{2}H_{T}L_{T}},$$

(2) $$C_{TZ}=\frac{F_{TZ}}{0.5\rho U^{2}B_{T}L_{T}},$$

(3) $$C_{TM}=\frac{M_{TX}}{0.5\rho U^{2}B_{T}^{2}L_{T}},$$

where *ρ* is the air density (1.225 kg/m^3^); *U* is the airflow speed; *L_T_* (length), *B_T_* (breadth) and *H_T_* (height) of the train are shown in Figure 3. In each experimental scenario, as the measurement of the wind pressure lasted for 20 s, these coefficients could be obtained as the time-averaged results. The side-force coefficient (*C_TY_*) and overturning-moment coefficient (*C_TM_*) were investigated in this research since they were more closely associated with the overturning safety of the train, which is the main concern for trains travelling in strong wind conditions. 

In the literature, the effectiveness of the wind barriers for the mitigation of wind effects was mainly evaluated in terms of the aerodynamic coefficients (e.g., *C_TY_*, *C_TZ_* and *C_TM_*) of the train due to the crosswind; meanwhile, the performance of the bridge was not taken into account. As understood, the barriers may present additional loading on the bridge and may result in further design requirements or risks for the bridge. In order to investigate the mechanical and aerodynamic effects of the wind barriers added to the bridge, the aerodynamic coefficients, including the side-force coefficient (*C_BY_*) and overturning-moment coefficient (*C_BM_*) for the bridge, were also defined and determined using Equations (4) and (5):

(4) $$C_{BY}=\frac{F_{BY}}{0.5\rho U^{2}H_{B}L_{B}},$$

(5) $$C_{BM}=\frac{M_{BX}}{0.5\rho U^{2}B_{B}^{2}L_{B}},$$

where the *L_B_* (length), *B_B_* (breadth) and *H_B_* (height) of the bridge are as shown in Figure 3. It was reported in [13] that the airflow speed may not obviously affect these aerodynamic coefficients for the train for a given height and porosity of the wind barrier. Therefore, the aerodynamic coefficients were measured at a specified airflow speed level, e.g., 10 m/s, according to [10]. However, since GFRP wind barriers with relatively low stiffnesses were used in this study, obvious deformation was expected when a crosswind was applied. As a result, the aerodynamic coefficients of the train and bridge system may change with the airflow speed due to the deformation in the shape of the wind barrier. Different levels of the airflow speed were therefore applied in the experiments and the aerodynamic coefficients were determined respectively.

## 4. Modelling of the Train–Barrier–Bridge System

### 4.1. Modelling Assumptions and Geometries

The aerodynamic performance of the bridge–train–barrier system may be affected by different dimensional parameters and material properties of the barrier, and it would be time-consuming, laborious and costly to optimise all these factors. In contrast, numerical modelling provides a method for conveniently investigating the influential factors that are able to bridge the gap between reduced- and full-scale investigations. Based on the configuration and results in the experimental investigation, reduced-scale (1:40) and a full-scale models were established in the numerical modelling using Ansys 17.0 (Ansys, Pittsburgh, PA, USA) for the train–barrier–bridge system. The wind barriers were defined with an elastic modulus of 8.6 GPa and a Poisson’s ratio of 0.183, as obtained by the tensile test. The bottom end of the barrier was fixed on the bridge and the top was a free end. The airflow condition and the deformation of the adaptive wind barrier made from GFRP were coupled together through the fluid–solid coupling model provided in Ansys. This meant that the displacement of the nodes in the wind barrier in the *y*- and *z*-directions (see Figure 3) due to the wind pressure would affect the airflow conditions in turn. The mechanical behaviours of the airflow, train and bridge in terms of the lift force, side force and overturning moment were solved using a Fluent module via a static analysis; meanwhile, the stress and deformation of the barrier were solved using a transient structural module via a transient analysis. Several assumptions were considered in the modelling. The friction between the airflow and all surfaces was neglected. In addition, the realizable *k*-*ε* turbulence model provided by Ansys was used for the airflow, where *k* is the turbulence kinetic energy and *ε* is the turbulent dissipation rate; the model is usually used for scenarios with mainly planar surfaces, as encountered in this case, and may offer acceptable convergence. The non-equilibrium wall function provided by Ansys for the modelling of the wall surface in contact with the flow was used for the barrier, as this function is often considered for simulations with rapid changes in the velocity and direction of the flow [26,27]. Finally, the compressibility of the air was neglected. 

The dimensions of the reduced-scale FE model was identical to the system examined in the wind tunnel experiments, as shown in Figure 3. The length of the train–barrier–bridge system was 2 m and the thickness of the wind barrier was 0.6 mm. The size of the computational field was 3 × 3 × 15 m (height × width × length). Four different barrier heights (i.e., 0, 8, 10 and 12 cm) were considered to provide an adequate validation of the modelling approach. For the full-scale FE model, the dimensions of the train and bridge were 40 times larger than those used in the reduced-scale modelling. For example, the length of the bridge became 80 m. Accordingly, the computational domain was also increased to 120 × 120 × 600 m (height × width × length).

### 4.2. Meshing and Boundary Conditions

**Figure 4:**
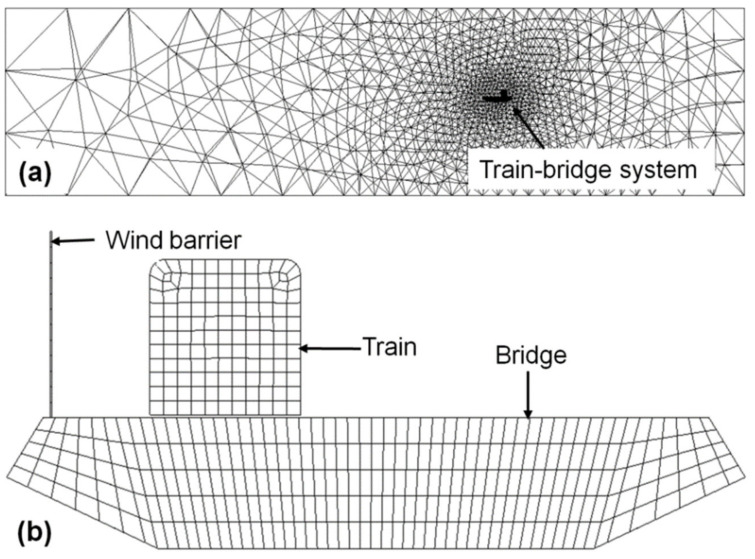
Meshing details for (**a**) the overall computational domain in the reduced-scale Fluent modelling and (**b**) the train–barrier–bridge system in the transient structural modelling.

The computational domain and the train–barrier–bridge system in the modelling were meshed individually, as shown in Figure 4a,b. Non-structural tetrahedral elements were used and the element size was approximately 1 cm for the train and approximately 1 to 3 cm for the bridge for both the full- and reduced-scale modelling. The elements for the airflow had an increased density as it approached the train–bridge system. For example, in the reduced-scale modelling, the element size was about 40 cm near the boundary of the computational domain and decreased to the same size as the bridge elements (1 to 3 cm) when approaching the train–barrier–bridge system. 

The airflow inlet boundary was defined by the velocity inlet function in the Fluent module, where the turbulence mode was defined by the intensity and length scale, as these were the characteristics that affected the turbulence according to actual testing conditions in the wind tunnel [16]. The outlet boundary was defined by the pressure inlet function with the same settings as the turbulence at the inlet boundary. The surfaces of the wind barrier were defined as coupled interfaces; meanwhile, the contact surfaces of the train, the bridge and the computational domain were defined as a wall without friction.

## 5. Experimental and Numerical Results

### 5.1. Deformation of the Wind Barrier Due to a Crosswind

**Figure 5:**
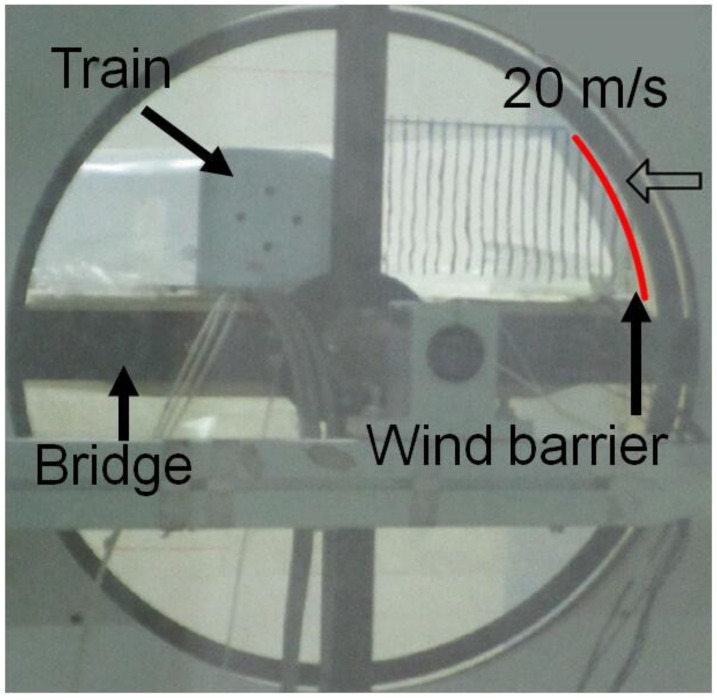
Deformed shapes of the wind barrier at an airflow speed of 20 m/s.

**Figure 6:**
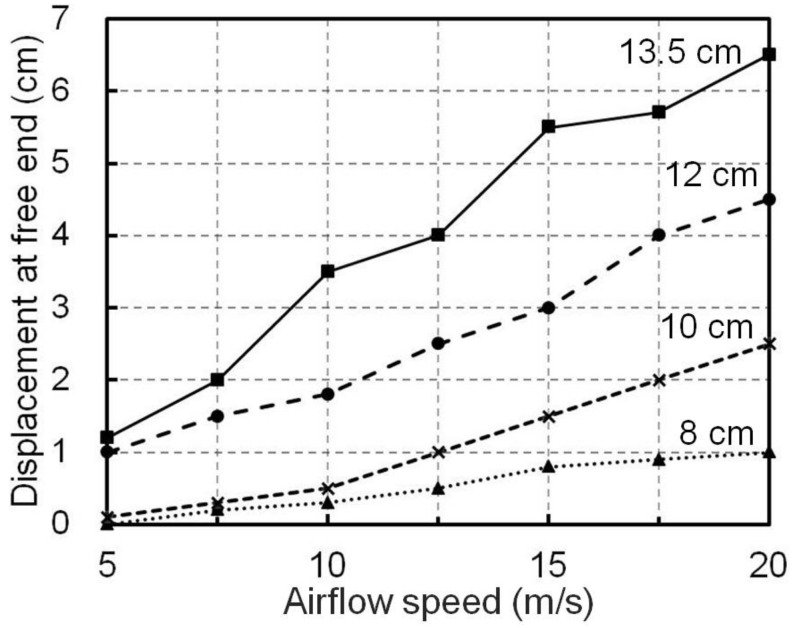
Displacement at the free end of the wind barrier with different heights subjected to crosswinds at different speeds.

Adaptive deformation of the wind barrier was witnessed in the wind tunnel experiments, as shown in the side view of the train–barrier–bridge system under the effect of a crosswind (Figure 5), where the barrier had a height of 13.5 cm and the train was placed in the leeward direction. When the speed of the airflow was 5 m/s in the experiments, the barrier was visually vertical but notable deformation was witnessed when the speed of the applied airflow was increased to 20 m/s, as shown in Figure 5. The lateral displacement at the free end of the wind barrier was measured and recorded to quantify the deformation of the barriers, as shown in Figure 6. It can be seen that the displacement clearly increased with the airflow speed and the barrier height. For example, the displacement was only 0.1 cm at the free end of the barrier with a height of 10 cm at an air flow of 5 m/s, but it increased to 2.5 cm when the airflow speed was 20 m/s. The displacement became more obvious for the 13.5 cm barrier, where the maximum displacement was 6.5 cm, which was recorded with an airflow speed of 20 m/s. 

### 5.2. Effects of the Presence and Location of the Train

**Figure 7:**
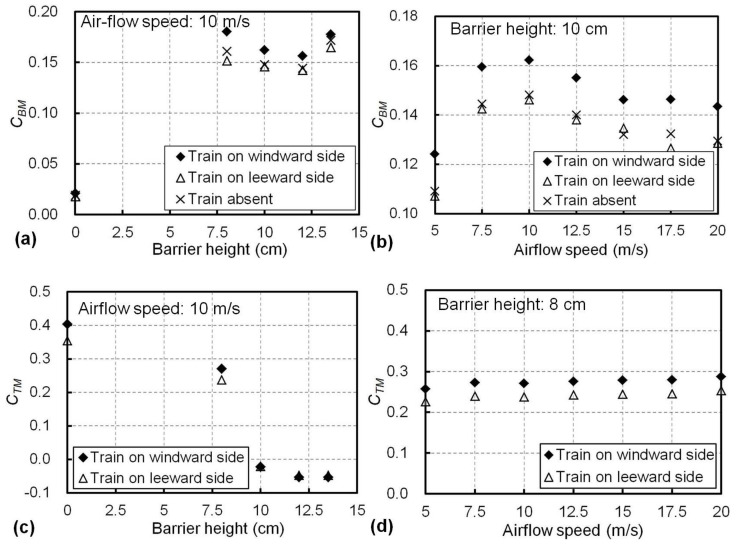
Overturning-moment coefficient responses for different train locations for (**a**) the bridge at an airflow speed of 10 m/s and different barrier heights, (**b**) the bridge with a barrier height of 10 cm and different airflow speeds, (**c**) the train at an airflow speed of 10 m/s and different barrier heights, and (**d**) the train with a barrier height of 8 cm and different airflow speeds.

The airflow may have different aerodynamic effects when the train is on the windward side or leeward side or absent. These three scenarios were investigated in the wind tunnel experiments, as shown in Figure 3, for different barrier heights and airflow speeds. The overturning-moment coefficient of the bridge (*C_BM_*) and the train (*C_TM_*) from the different experimental scenarios are presented in Figure 7. The change in *C_BM_* with the barrier height from 0 to 13.5 cm is shown in Figure 7a for a constant airflow speed of 10 m/s. It can be seen in Figure 7a that when the wind barrier was not used (i.e., the barrier height was 0 cm), *C_BM_* was almost identical for the three scenarios with a value of approximately 0.02. When barriers with the heights of 8, 10, 12 and 13.5 cm were used, *C_BM_* significantly increased to over 0.14 and varied between 0.14 to 0.18. For all the barrier heights, the bridge with a windward train was associated with the maximum *C_BM_* values in the three scenarios, as evidenced in Figure 7a. 

The *C_BM_* results at different airflow speeds with a constant barrier height of 10 cm are presented in Figure 7b. For all the airflow speeds applied in the experiments, the *C_BM_* of the bridge with a train on the windward side was higher than the other scenarios. Similar *C_BM_* values were witnessed when the train was absent or on the leeward side. For example, at an airflow speed of 10 m/s, *C_BM_* was 0.162 when the train was on the windward side, where this was greater than those of the bridge with a leeward train (0.146) or without a train (0.148). 

The effects of the barrier height on the overturning-moment coefficient (*C_TM_*) of the train in the windward or leeward side are illustrated in Figure 7c. When an 8 cm barrier was used, *C_TM_* significantly decreased from more than 0.36 (no wind barrier, see Figure 7c) to less than 0.27 for both the windward and leeward sides. For barriers taller than 10 cm, *C_TM_* became close to 0 or even negative, as shown in Figure 7c, indicating only small overturning moments were imparted to the train and a negative value means the moment was in the direction opposite to the moment illustrated in Figure 3. At an airflow speed of 10 m/s, a train in the windward side presented a higher value of *C_TM_*, as shown in Figure 7c. Meanwhile, as presented in Figure 7d, when an 8 cm barrier was used, the change in *C_TM_* with the airflow speed was insignificant, where the *C_TM_* values ranged from 0.257 to 0.287 for the windward train and 0.226 to 0.253 for the leeward one. Again, the windward train always presented a higher C_TM_ value in Figure 7d.

Based on the above findings, in terms of the aerodynamic coefficients of the train and the bridge, the system with a windward train may be more critical for the train and the bridge because of the higher values of the associated aerodynamic coefficients (*C_TM_* and *C_BM_*). Therefore, this scenario was further focused upon in the following analysis, and the results from the other two scenarios are not included.

### 5.3. Effects of the Barrier Height and the Airflow Speed on the Aerodynamic Coefficients of the Train

**Figure 8:**
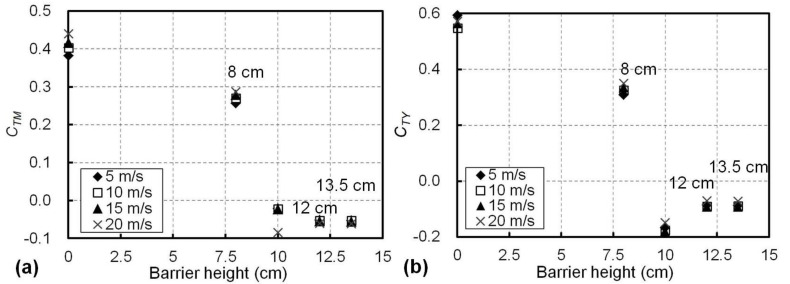
Aerodynamic responses of the train with different barrier heights at different airflow speeds for (**a**) the overturning-moment coefficient, *C_TM_*, and (**b**) the side-force coefficient, *C_TY_*.

The shielding effects of the proposed wind barriers with different heights were evaluated using the overturning-moment coefficient (*C_TM_*) and the side-force coefficient (*C_TY_*) of the train on the windward side, subjected to the crosswind with different speeds ranging from 5 to 20 m/s from the experiments. Figure 8a shows the effects of the barrier height and the airflow speed on *C_TM_*. For a given barrier height, the *C_TM_* values were similar at different airflow speeds, with a maximum variation within 0.07. Therefore, the effect of the airflow speed in the range of 5 to 20 m/s on the overturning-moment coefficient *C_TM_* of the train was therefore minor. However, *C_TM_* changed significantly with the barrier height. For example, *C_TM_* was 0.41 on average for different airflow speeds when the barriers were not used (i.e., the barrier height was 0 cm); meanwhile, it decreased to 0.27 on average when a barrier with a height of 8 cm was used (see Figure 8a). For barriers taller than 10 cm, the absolute values of *C_TM_* were within only 0.08, suggesting prominent shielding effects resulted in a minor overturning moment on the train.

The effects of the barrier height and airflow speed on side-force coefficient (*C_TY_*) of the train are presented in Figure 8b. Similar to their effects on *C_TM_*, a barrier taller than 8 cm significantly reduced the value of *C_TY_* from 0.57 (no wind barriers) to less than 0.19 (absolute value). For a given barrier height, the effect of the airflow speed on *C_TY_* was minor and the variation in *C_TY_* at different airflow speeds was within 0.05, as shown in Figure 8b. Therefore, according to the *C_TY_* and *C_TM_* results presented in Figure 8a,b, for a favourable result on the train in the experiments, a barrier taller than 10 cm achieved superior shielding effects, with the average absolute values of 0.04 for *C_TM_* and 0.17 for *C_TY_*. In addition, since both *C_TY_* and *C_TM_* were not sensitive to the airflow speeds in the range of 5 to 20 m/s, it may be practical to apply a constant airflow speed in the wind tunnel experiments for the determination of *C_TY_* and *C_TM_*, as is usually conducted in the literature [6,8,28].

### 5.4. Effects of the Barrier Height and Airflow Speed on the Aerodynamic Coefficients of the Bridge

**Figure 9:**
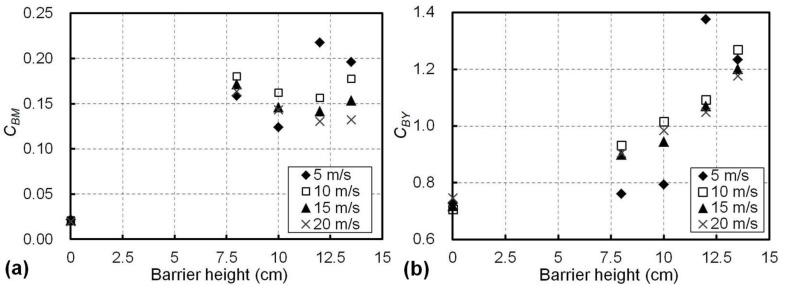
Aerodynamic responses of the bridge with different barrier heights at different airflow speeds for (**a**) the overturning-moment coefficient, *C_BM_*, and (**b**) the side-force coefficient, *C_BY_*.

The responses of the bridge with five different barrier heights (i.e., 0, 8, 10, 12 and 13.5 cm) to different airflow speeds (5, 10, 15 and 20 m/s) are presented in Figure 9a,b for the overturning-moment coefficient (*C_BM_*) and the side-force coefficient (*C_BY_*). As shown in Figure 9a, *C_BM_* was only 0.02 for the bridge without a barrier for all applied airflow speeds, while it increased sharply to more than 0.15 when a barrier was used with a height of 8 cm. This meant the barriers introduced an adverse mechanical effect to the bridge in terms of the overturning moment. According to Figure 9a, when the barriers were used, *C_BM_* presented a non-linear relation relative to the barrier height, as its value reduced from 0.169 to 0.144 when the barrier height increased from 8 cm to 10 cm, which was then maintained within a range of 0.162 to 0.165 for the barrier height from 12 to 13.5 cm for all the applied airflow speeds. 

As shown in Figure 9b, for the airflow speeds from 10 to 20 m/s, the side-force coefficient (*C_BY_*) clearly increased with the barrier height. For example, when an 8 cm barrier was used, the average *C_BY_* increased from 0.72 (no wind barrier) to 0.91, and when the barrier height increased from 12 cm to 13.5 cm, *C_BY_* increased sharply from 1.07 to 1.22. Therefore, a barrier with a smaller height introduced a smaller side force on the bridge. In comparison to the barrier heights, the airflow speeds presented less of an effect on *C_BY_*, as the variation in *C_BY_* was within 0.1 when different airflow speeds (10, 15 or 20 m/s) were applied.

**Figure 10:**
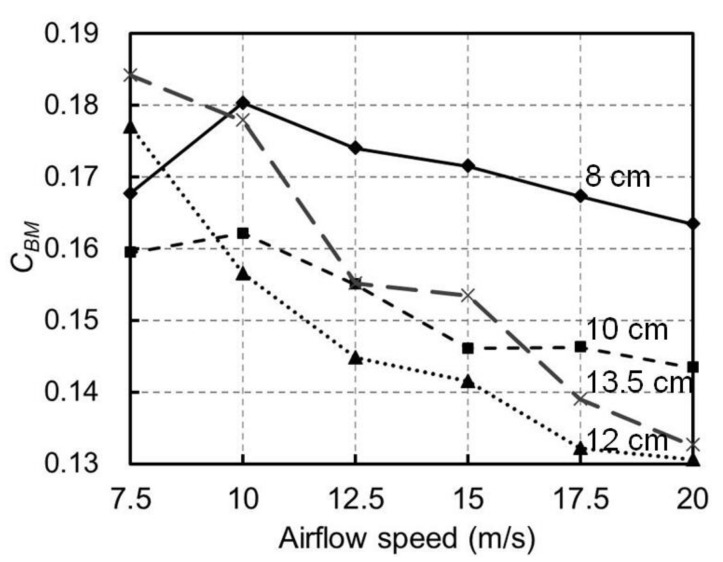
Effects of the airflow speeds on the overturning-moment coefficient *C_BM_* of the bridge with different wind barrier heights.

The changes in *C_BM_* with the airflow speeds are presented in Figure 10. It can be seen that, in general, *C_BM_* decreased with the increase of airflow speed above 10 m/s for all the tested barriers and such decreases in *C_BM_* with the airflow speed were more significant for taller barriers. For example, when the airflow speed increased from 10 to 20 m/s, *C_BM_* decreased by 9.4% (i.e., from 0.180 to 0.163) for the 8 cm barrier and by 25.2% (i.e., from 0.178 to 0.133) for the 13.5 cm barrier. This was possibly because the deformation of the barriers increased with their heights and airflow speeds; therefore, the barrier with the larger deformation was associated with the smaller wind resistance. In comparison to the wind barriers in the literature [13,14,15], with almost constant *C_BM_* and *C_BY_*, the proposed adaptive wind barriers with a proper height can therefore mitigate the loads and aerodynamic effects of a strong crosswind on the bridge via the reduction of its aerodynamic coefficients. Therefore, by considering the provided small aerodynamic coefficients of the train (see Figure 8) and the decrease in *C_BM_* with the increase of the airflow speed (see Figure 10), the adaptive wind barrier with a proper height helps to mitigate the crosswind effects on the trains in operation; meanwhile, in strong wind scenarios, it may still introduce lower mechanical loads on the bridge compared to existing wind barriers.

It is valuable to compare the aerodynamic coefficients obtained from this study to those received through the wind tunnel experiments on other types of wind barriers, where a bridge with the same cross-section was used [16]. Three other types of wind barriers (i.e., fence-type, louver-type and grid-type) were considered with a height of 7.5 cm and a porosity of 26% [16]. The overturning-moment coefficient (*C_BM_*) and the side-force coefficient (*C_BY_*) of the bridge from [16] are listed in Table 1, together with the corresponding experimental results from the model bridge with an 8 cm barrier in this study. It can be seen from Table 1 that smaller values of *C_BM_* and *C_BY_* were achieved by the proposed wind barrier in this study in comparison to the fence-type and grid-type barriers, where this means a smaller overturning moment and side force transferred from wind barriers to the bridge. The overturning-moment coefficient (*C_BM_*) offered by the louver-type barrier was 0.49 and 47% smaller than that from the proposed adaptive barrier, suggesting that a smaller moment was transferred by the louver-type barrier. The side-force coefficient (*C_BY_*) was 0.16, i.e., 11% less than the proposed adaptive barrier, suggesting a similar aerodynamic performance in terms of the side-force transfer. However, the blades of the louver-type barrier need to be adjusted for their angles to accommodate different wind scenarios; therefore, it has essential requirements of power and an active control system during operations and maintenance, as mentioned before. 

### 5.5. Results from the Reduced- and Full-Scale Modelling

In order to further demonstrate the practicability of the proposed adaptive wind barriers at full scale, FE modelling for the reduced-scale train–barrier–bridge system was developed using Ansys for validation through a comparison to the wind tunnel experiments. The modelling approach could then be used to build a full-scale model (i.e., 40 times the dimension of the tested specimen) to investigate the aerodynamic performance of the train–barrier–bridge system at full scale, especially considering that the aerodynamic coefficients obtained from reduced-scale experiments may not be directly used for large scale structures in practice [29,30].

**Figure 11:**
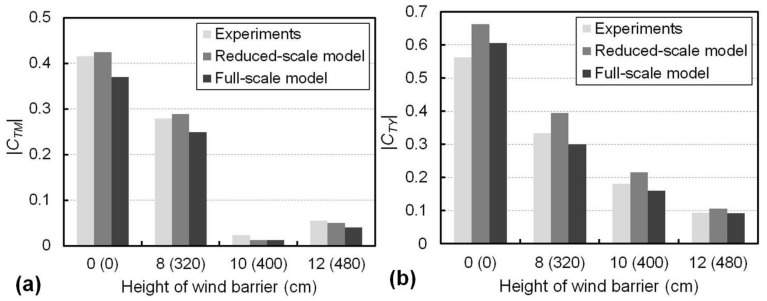
Comparison between the experimental and numerical results for (**a**) *C_TM_* and (**b**) *C_TY_* with different wind barrier heights. The heights in brackets correspond to the heights in the full-scale model.

The values of both the overturning-moment coefficient (*C_TM_*) and side-force coefficient (*C_TY_*) for the train at an airflow speed of 15 m/s from the reduced- and full-scale modelling, together with the results from the experiments, are presented in Figure 11. The horizontal axis gives the height of the barriers, with the numbers in brackets being the barrier heights in the full-scale model. As shown in Figure 11a, the *C_TM_* values were consistent between the experiments and the reduced-scale modelling, and they were greater than that from the full-scale modelling. For example, *C_TM_* for the 8 cm barrier was 0.28 from the experiments and 0.29 from the reduced-scale modelling, while *C_TM_* for the 320 cm barrier in the full-scale modelling was 0.25. The favourable shielding effect of a barrier taller than 10 cm (i.e., 400 cm in the full-scale model) was supported by the smaller values of *C_TM_* from the modelling results at both the reduced and full scales, as evidenced by the significant decreases of the *C_TM_* values shown in Figure 11a. The values of the side-force coefficient of the train (*C_TY_*), shown in Figure 11b, indicate that *C_TY_* decreased with the barrier height for both the reduced- and full-scale approaches with the maximum variation being within 25%. Nevertheless, the validated FE modelling approach is suggested as an effective way to understand the aerodynamic performance of the train–barrier–bridge system at full scale.

**Figure 12:**
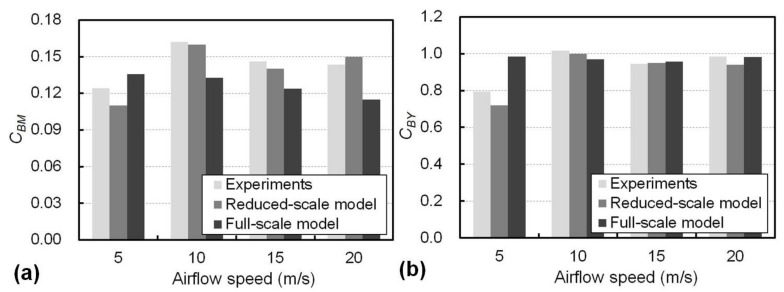
Effects of the airflow speeds on (**a**) *C_BM_* and (**b**) *C_BY_*. based on the wind tunnel experiments and modelling.

The results of overturning-moment coefficient of bridge, *C_BM_*, for different airflow speeds from the wind tunnel experiments and from both the reduced- and full-scale modelling are presented in Figure 12a, where the barrier height of 10 cm in the experiments corresponds to 400 cm in the full-scale modelling. The *C_BM_* values decreased with the increase in airflow speed, which is consistent with those previously presented in Figure 10, and such a decrease became prominent when the airflow speed was over 10 m/s due to the smaller resistance to wind from the barrier after its deformation. Furthermore, the decrease in *C_BM_* was more obvious in the full-scale model in comparison to the reduced-scale wind tunnel experiments; therefore, the mitigation in the transfer of wind load to the bridge by the proposed adaptive wind barrier may be valid in practice. The effects of the airflow speeds on *C_BY_* are presented in Figure 12b, where the variation in *C_BY_* from the experiments and the full-scale modelling for the barrier with a height of 10 cm was within 4% for airflow speeds from 10 to 20 m/s, suggesting the effect of the airflow speed on the side-force coefficient of the bridge in this range was not significant.

## 6. Conclusions

In this study, an innovative concept for a wind barrier was proposed to reduce the wind load transferred from the barrier to the bridge through the adaptive deformation of the barrier in crosswind scenarios. The barriers were made of GFRP composites with a relatively low bending stiffness, therefore allowing them to deform by bending more prominently in comparison to traditional wind barriers made from steel and reinforced concrete. Reduced-scale wind tunnel experiments were conducted to investigate the effects of the barriers on the aerodynamic performance of the train and bridge, including their side-force and overturning-moment coefficients. A reduced-scale FE model was developed and its results were validated by comparing them with the experimental results. The validated modelling approach was then used to develop a full-scale train–barrier–bridge system to understand the mechanical and aerodynamic characteristics in practice. The following conclusions may be drawn from this work:

1. The proposed wind barriers made from GFRP composites showed adaptive bending deformation when subjected to crosswind and more significant lateral deformation was seen with higher airflow speeds. Based on the results from the wind tunnel experiments, it can be seen that when the train was on the windward side on the bridge, both the train and the bridge were associated with a higher side-force and overturning-moment coefficients in comparison to the scenarios with the train in the leeward side or absent. For example, with a constant airflow speed of 10 m/s and a barrier height of 10 cm, the overturning-moment coefficient for the bridge (*C_BM_*) was 0.162 when the train was on the windward side, while it became less than 0.148 for the other two scenarios.
2. The wind tunnel experiments further showed that a wind barrier taller than 10 cm reduced the overturning-moment coefficient (*C_TM_*) of the train from 0.41 (without the wind barrier) to be less than 0.08, and reduced the side-force coefficient (*C_TY_*) of the train from 0.57 (without the wind barrier) to be less than 0.19. Therefore, the transfer of the wind load to the train could be effectively mitigated in this way. The values of *C_TM_* and *C_TY_* were not noticeably decreased when the barrier height was further increased from 10 cm to 13.5 cm. This suggests there may be an optimal height for the proposed wind barrier in terms of the aerodynamic performance of the train. Furthermore, when different airflow speed levels from 5 to 20 m/s were applied, the variations of *C_TM_* and *C_TY_* were insignificant, for example, within 10.5% for the former and 9.4% for the latter when the barrier height was 8 cm.
3. The wind tunnel experiments also indicated that for an airflow speed over 10 m/s, the proposed wind barrier increased the overturning-moment coefficient (*C_BM_*) of the bridge from 0.02 (without the wind barrier) to 0.15 (8 cm wind barrier) and increased the side-force coefficient (*C_BY_*) of the bridge from 0.72 (no wind barrier) to 0.97 (8 cm wind barrier). Again, the effects of a change in the airflow speeds on *C_BY_* was minor, while *C_BM_* significantly decreased when the airflow speed rose from 10 to 20 m/s for all barrier heights applied. Such a decrease was more obvious for a taller barrier, for example, 9.4% for the 8 cm barrier and 25.2% for the 13.5 cm barrier. This was in association with the increases of deformation of the barriers with their heights and airflow speeds; therefore, the barrier with a larger deformation showed less resistance to wind and then transferred lower loads to the bridge.
4. The results from the reduced-scale FE modelling were consistent with those from the wind tunnel experiments. The validated FE approach was used to develop the full-scale train–barrier–bridge system. The full-scale modelling results indicated that the change in *C_BY_* with airflow ranging from 5 to 20 m/s was not obvious, with a variation of only 4%. However, the decrease in the overturning-moment coefficient of the bridge, *C_BM_*, with the increase in the airflow speed was more prominent than the results from the wind tunnel experimental results. This suggests that the mitigation in the transfer of the wind load to the bridge by the proposed adaptive wind barrier was effective at the full scale in practice.

## Figures and Tables

**Table 1:** Aerodynamic coefficients of the bridge with different types of wind barriers.

Aerodynamic Coefficient	Fence-Type	Grid-Type	Louver-Type	Present Research
Moment Coefficient, *C_BM_*	1.13	1.13	0.49	0.93
Side-force Coefficient, *C_BY_*	0.29	0.31	0.16	0.18

## Data Availability

The raw/processed data required to reproduce these findings cannot be shared at this time as the data also forms part of an ongoing study.
